# Laparoscopic Repair of Internal Transmesocolic Hernia of Transverse Colon

**DOI:** 10.1155/2015/853297

**Published:** 2015-07-12

**Authors:** Tomokazu Kishiki, Toshiyuki Mori, Yoshikazu Hashimoto, Hiroyoshi Matsuoka, Nobutsugu Abe, Tadahiko Masaki, Masanori Sugiyama

**Affiliations:** Department of Surgery, Kyorin University School of Medicine, 6-20-2 Shinkawa, Mitaka City, Tokyo 181-8611, Japan

## Abstract

*Introduction*. Internal hernias are often misdiagnosed because of their rarity, with subsequent significant morbidity. *Case Presentation*. A 61-year-old Japanese man with no history of surgery was referred for intermittent abdominal pain. CT suggested the presence of a transmesocolic internal hernia. The patient underwent a surgical procedure and was diagnosed with transmesocolic internal hernia. We found internal herniation of the small intestine loop through a defect in the transverse mesocolon, without any strangulation of the small intestine. We were able to complete the operation laparoscopically. The patient's postoperative course was uneventful and the patient was discharged on postoperative day 6. *Discussion*. Transmesocolic hernia of the transverse colon is very rare. Transmesocolic hernia of the sigmoid colon accounts for 60% of all other mesocolic hernias. Paraduodenal hernias are difficult to distinguish from internal mesocolic transverse hernias. We can rule out paraduodenal hernias with CT. *Conclusion*. The patient underwent a surgical procedure and was diagnosed with transmesocolic internal hernia. We report a case of a transmesocolic hernia of the transverse colon with intestinal obstruction that was diagnosed preoperatively and for which laparoscopic surgery was performed.

## 1. Introduction

Transmesocolic hernia is an extremely rare type of internal hernia [1]. The reported incidence of internal hernias ranges from 0.6% to 5.8% of all small bowel obstructions [2], and transmesocolic hernia accounts for approximately 5% to 10% of all internal hernias [3].

Transmesocolic hernia of the transverse colon is very rare. Transmesocolic hernia of the sigmoid colon accounts for 60% of all other mesocolic hernias [4].

Ordinarily, hernias develop in a preexisting anatomical orifice such as the foramen of Winslow [5]. Congenital anomalies due to improper intestinal rotation, previous trauma, vascular or inflammatory diseases, and postsurgical iatrogenic conditions are factors that predispose patients to internal herniation [6].

Internal abdominal hernia has a nonspecific and intermittent clinical presentation; therefore, presurgical diagnosis is rare [7]. Its diagnosis remains difficult, even after utilization of computed tomography (CT) [5, 6].

We report a case of a transmesocolic hernia of the transverse colon with intestinal obstruction that was diagnosed preoperatively and for which laparoscopic surgery was performed.

## 2. Case Report

**Figure 1:**
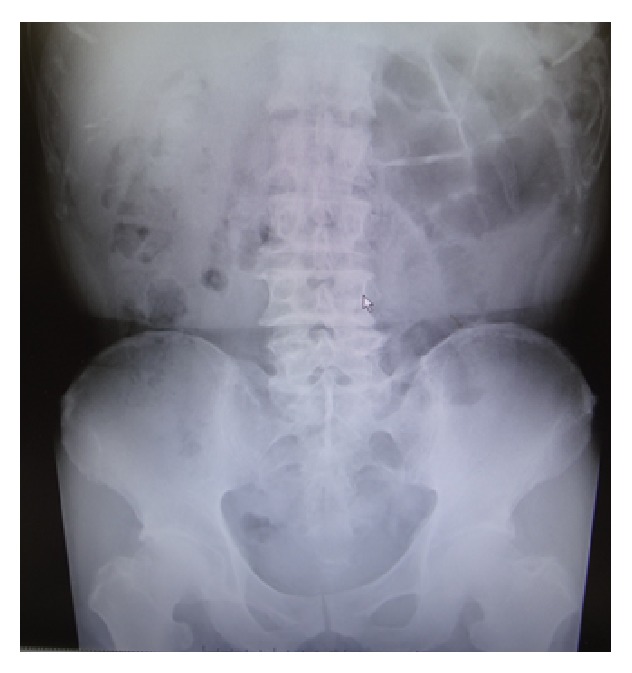
A radiograph shows the dilated small bowel in the upper quadrant.

**Figure 2:**
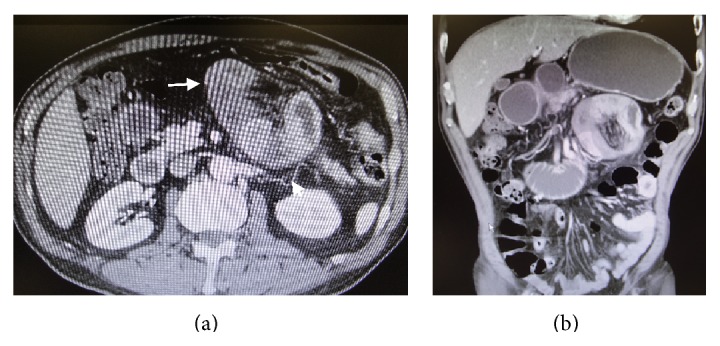
An axial computed tomography scan shows an encapsulated cluster of dilated small bowel loops (arrow) occupying the upper quadrant. A small bowel loop is ascending the left colic artery (arrowhead).

**Figure 3:**
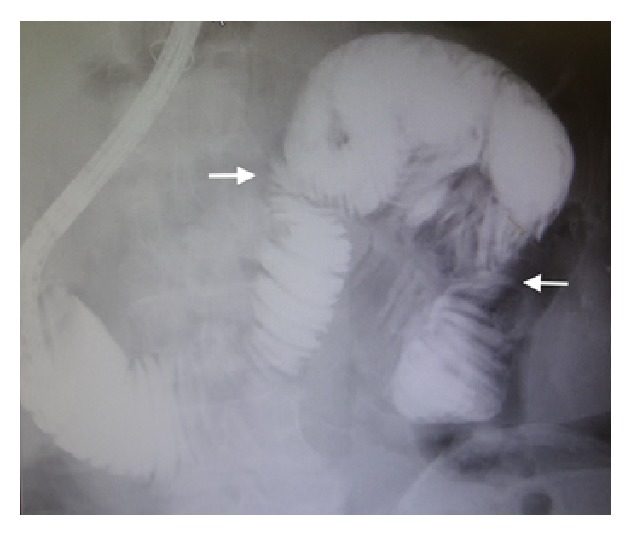
A gastrointestinal series with barium contrast showing dilated loops of the small bowel in the upper quadrant, delay of contrast, or the point of obstruction (arrow).

A 61-year-old Japanese man was admitted to our hospital with intermittent abdominal pain. He had no past history of abdominal surgery. His vital signs were stable. On examination, the abdomen was soft and nontender. Laboratory investigation measurements on admission were normal. An abdominal radiograph showed the air-fluid levels in the upper quadrant with no free air under the dome of the diaphragm (Figure 1). An enhanced CT scan of the abdomen showed clustered encapsulated and dilated small bowel loops predominantly in the middle abdomen (Figures 2(a) and 2(b)). The inferior mesenteric vein (IMV) and ascending left colic artery were behind the small bowel loops. On Gastrografin contrast examination using double balloon-assisted endoscopy, he was found to have an incomplete stricture (Figure 3).

These findings suggested the presence of an internal hernia excluding paraduodenal hernias. The patient underwent a surgical procedure and was diagnosed with transmesocolic internal hernia.

**Figure 4:**
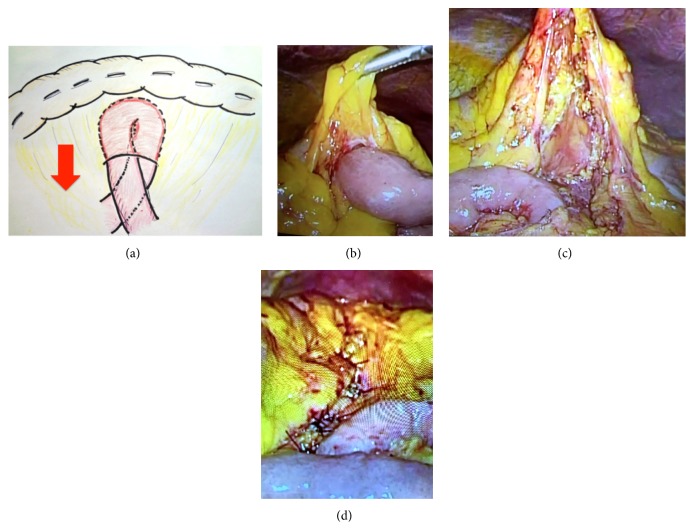
*Operative findings*. (a) Illustration. (b) The ileum is herniated through the mesenteric defect. (c) The small intestine is pulled out through the mesenteric defect. (d) Closure of the mesenteric defect.

We chose to treat the obstruction laparoscopically with a four-trocar approach. Intraoperatively, we found internal herniation of the small intestine loop through a defect in the transverse mesocolon, without any strangulation of the small intestine (Figure 4(a)). The herniated small intestine could be loosened and retracted back through the hernia (Figure 4(b)). The defect was only in the posterior mesenteric membrane. The mesenteric anterior membrane formed the hernial sac (Figure 4(c)). The defect was sutured by using the anterior and posterior mesenteric membranes (Figure 4(d)). We were able to complete the operation laparoscopically.

The patient's postoperative course was uneventful and the patient was discharged on postoperative day 6. At the 1-year follow-up examination, no clinical or radiographic evidence of the internal hernia was observed.

## 3. Discussion

An internal hernia is defined as the protrusion of viscus through a normal or abnormal opening within the confines of the abdominal cavity [8]. These hernias may be either congenital or acquired. The herniation may be persistent. Few cases of mesocolic hernia are reported [9]. Among adults, the main causes of internal hernias are previous gastrointestinal surgery, abdominal trauma, or intraperitoneal inflammation [5, 10, 11]. Our case of internal hernia was a rare presentation in an adult without a history of trauma or previous surgery.

A transmesocolic hernia is difficult to diagnose preoperatively and often requires resection of the affected intestine [2, 5]. While some patients with internal hernias are asymptomatic, others have nonspecific symptoms such as chronic dyspepsia, intermittent colicky abdominal pain, and vomiting. The rare occurrence of internal hernias (5.8% of all small bowel obstructions) [12] and the absence of specific clinical features make a clinical diagnosis difficult [9]. The laboratory findings are frequently inconclusive.

Internal hernias are often misdiagnosed because of their rarity, with subsequent significant morbidity. Paraduodenal hernias are difficult to distinguish from internal mesocolic transverse hernias. Paraduodenal hernias originate at the fossa of Landzert, which is located immediately lateral to the fourth segment of the duodenum and behind the IMV and ascending left colic artery. We can rule out paraduodenal hernias with CT. CT is the gold standard test to diagnose internal abdominal hernia. CT findings may include displacement of the mesenteric trunk toward the hernia, elongation, grouping and engorgement of the mesenteric vessels, abnormal encapsulation of intestinal loops in the peritoneal cavity, stasis, and absence of intraluminal contrast progression associated with distension content [10, 11]. However, transmesenteric hernia cannot always be definitely diagnosed by CT. Abdominal radiography can provide information regarding the intestinal segment involved and the extension of the intestinal obstruction; a gastrointestinal series with barium contrast may show dilated loops of small bowel in the upper quadrant, delay of contrast, or the point of obstruction [5, 9].

There are few reported cases of laparoscopic repair of internal transmesocolic hernia [13]. Laparoscopic repair would be expected to reduce postoperative pain, morbidity, and length of hospital stay. Additionally, the laparoscopic approach has the abilities of both diagnosis verification and simultaneous surgical intervention in cases in which internal hernia is suspected but cannot be confirmed by preoperative imaging studies. During the urgent intervention, the laparoscopic approach is more difficult for bowel loop distensions that may reduce the operative space and hamper surgical movements with more risk of lesions [14]. In our department, laparoscopy is indicated when there are no signs of bowel necrosis or dilatation of the incarcerated bowel loops.

## 4. Conclusion

The internal transmesocolic hernia is a rare intestinal obstruction that should be considered in cases of recurrent abdominal pain and obstruction without any history of surgical intervention, external hernia, or inflammatory disease. As in our case, the early diagnosis can reduce complications such as obstruction, necrosis, and perforation. Surgical intervention is the treatment of choice, particularly in asymptomatic cases, because it reduces the urgent surgery and complications associated with hernia. The laparoscopic approach, although more difficult to perform as an emergency procedure, is considered for cases such as ours, because it can reduce morbidity, postoperative pain, and length of hospital stay.
